# Exclusively Relativistic: Periodic Trends in the Melting and Boiling Points of Group 12

**DOI:** 10.1002/anie.202100486

**Published:** 2021-02-26

**Authors:** Jan‐Michael Mewes, Peter Schwerdtfeger

**Affiliations:** ^1^ Mulliken Center for Theoretical Chemistry University of Bonn Beringstrasse 4 53115 Bonn Germany; ^2^ Centre for Theoretical Chemistry and Physics The New Zealand Institute for Advanced Study Massey University Auckland 0632 Auckland New Zealand

**Keywords:** free-energy calculations, Group 12, phase transitions, relativistic effects, *λ*-scaling

## Abstract

First‐principles simulations can advance our understanding of phase transitions but are often too costly for the heavier elements, which require a relativistic treatment. Addressing this challenge, we recently composed an indirect approach: A precise incremental calculation of absolute Gibbs energies for the solid and liquid with a relativistic Hamiltonian that enables an accurate determination of melting and boiling points (MPs and BPs). Here, we apply this approach to the Group 12 elements Zn, Cd, Hg, and Cn, whose MPs and BPs we calculate with a mean absolute deviation of only 5 % and 1 %, respectively, while we confirm the previously predicted liquid aggregate state of Cn. At a non‐relativistic level of theory, we obtain surprisingly similar MPs and BPs of 650±30 K and 1250±20 K, suggesting that periodic trends in this group are exclusively relativistic in nature. Ultimately, we discuss these results and their implication for Groups 11 and 14.

## Graphical abstract

Only very recently has it become possible to obtain accurate melting and boiling points from quantum‐chemical simulations. These show that the periodic trends observed for the Group 12 elements are exclusively relativistic in nature, which can be rationalized in terms of the relativistic change in the corresponding cohesive energies.

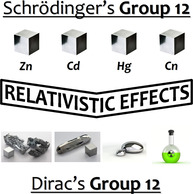

## Introduction

Home to the only liquid metal and sandwiched between the transition metals and main‐group elements, Group 12 (Zn, Cd, Hg, Cn) and its periodic trends are a prominent topic in science as well as in chemistry education. These elements are often used to illustrate the relativistic inert‐pair effect, as well as to discuss the surprising contrast between the important biological role of Zn vs. the highly toxic nature of Cd and Hg.^[1]^ Also from an electronic structure viewpoint, the interactions between these closed‐shell elements are interesting and manifold, ranging from van‐der‐Waals‐like behavior in small clusters, to covalent bonding at medium size, and finally metallic character at large cluster size continuing into the bulk.[^2^, ^3^, ^4^] This drastic change in bonding behavior with increasing cluster size is in accordance with very large changes in bond distances *r*_e_ and binding energies *D*_e_ from the dimers (*r*_e_/*D*_e_ are 3.83 Å/−0.03 eV for Zn_2_, 3.87/−0.04 for Cd_2_, 3.68/−0.05 for Hg_2_)^[5]^ to the bulk metals (*r*_e_/*E*_coh_ are 2.66 Å/−1.35 eV for Zn, 2.98/−1.16 for Cd, 3.01/−0.67 for Hg).[^6^, ^7^]

Already the evolution of the cohesive energy of the Group 12 metals shows a clear trend of increasing inertness from the lighter to the heavier elements. In Hg, relativistic effects are particularly strong due to their general scaling with *Z* ^2^. Specifically, the strong relativistic contraction and stabilization of the 6s shell renders Hg chemically inert, which is further enhanced by the soft and polarizable underlying 5d shell.[^9^, ^10^, ^11^] This is evident, e.g., from the relativistic‐to‐non‐relativistic ratio for the 6s orbital binding energy of 1.257.^[12]^ To a far lesser extent, the lanthanide contraction originating from the poor shielding of the nuclear charge by the filled 4f shell contributes to these effects.^[13]^ Altogether, the impact of relativistic effects gives rise to a manifold of peculiar properties of Hg, most prominent perhaps the low MP of 234.3 K,[^14^, ^15^] but also its unusually high superconducting‐transition temperature (*T*_c_=4.15 K) compared to Zn (*T*_c_=0.86 K) or Cd (*T*_c_=0.52 K),[^16^, ^17^] the appearance of an unusual oxidation state +4,^[18]^ and the chain‐like cinnabar crystal structure adopted by HgS.^[19]^ Furthermore, a shift of the appearance of a metallic state in clusters of increasing size has also been traced back to the impact of relativity.^[20]^ This renders it almost impossible to predict the physical and chemical behavior of the heavier Group 12 element Hg purely from periodic trends as originally proposed by Mendeleev.^[21]^

Moving even further down in the periodic table, Cn (*Z*=112) is the latest addition to Group 12, and with an α‐decay half‐life of 29 s for ^285^Cn one of the longest‐lived super‐heavy elements.[^22^, ^23^, ^24^] Its lifetime is sufficient to explore periodic trends through atom‐at‐a‐time experiments, which have provided an estimate for the cohesive energy of −0.4±0.1 eV.[^25^, ^26^, ^27^] This value agrees nicely with a recent high‐level theoretical (coupled‐cluster) value of −0.38±0.03 eV.^[15]^ In Cn, the relativistic contraction of the 7s shell and the expansion of the 6d_5/2_ shell are eventually so strong that they lead to a reversal in the energetic ordering of these levels, which has important consequences for its properties and chemical nature. Although it might, in contrast to all other members in this group, be regarded as a “real” d‐block element,^[28]^ the s–d inversion suppresses any metallic character in the bulk and effectively prevents covalent bonding.^[29]^ This leads to an increased chemical inertness reflected in the small cohesive energy and an almost noble‐gas like behavior.[^15^, ^26^, ^30^, ^31^] Accordingly, relativistic DFT/PBEsol and self‐consistent GW calculations have recently predicted that the Cn is an insulator with a rather large bandgap of 6.4 eV and a liquid at ambient conditions,^[31]^ confirming an almost 50 years old prediction by Pitzer.^[29]^ This has renewed the interest in the periodic trends of the MPs and BPs of the Group 12 elements.

To provide a discussion of these periodic trends with a quantitative basis, we report a comprehensive investigation of the influence of relativistic effects on their MPs and BPs. To this end, we first establish that our free‐energy‐based approach combined with a spin–orbit relativistic density functional theory (DFT) Hamiltonian can accurately recover the experimentally known MPs and BPs. Subsequently, we explore the influence of spin–orbit coupling and scalar‐relativistic effects through additional calculations in the non‐relativistic limit, and eventually revisit a previous prediction for Cn with a recently presented and more adept methodology.[^30^, ^31^]

## Results and Discussion

Before we start the discussion of our Gibbs‐energy calculations, let us set the stage by briefly reviewing the electronic structure and bonding of the Group 12 metals, as well as some general trends regarding the MP, BP, and cohesive energy. At the Hartree–Fock level, none of the Group 12 dimers are bound, or in other words, the chemical bonding is correlation driven,[^32^, ^33^, ^34^] as one would expect for dispersion‐bound systems. This propagates into the solid as shown, for example, for Hg by Paulus and co‐workers.[^34^, ^35^, ^36^] As a consequence, without electron correlation, all Group 12 elements would be gaseous at normal conditions, much like the noble gases, and only under pressure, they would become metallic. The important question is thus how relativistic effects impact the correlation‐driven bonding in the condensed phases.

**Figure 1:**
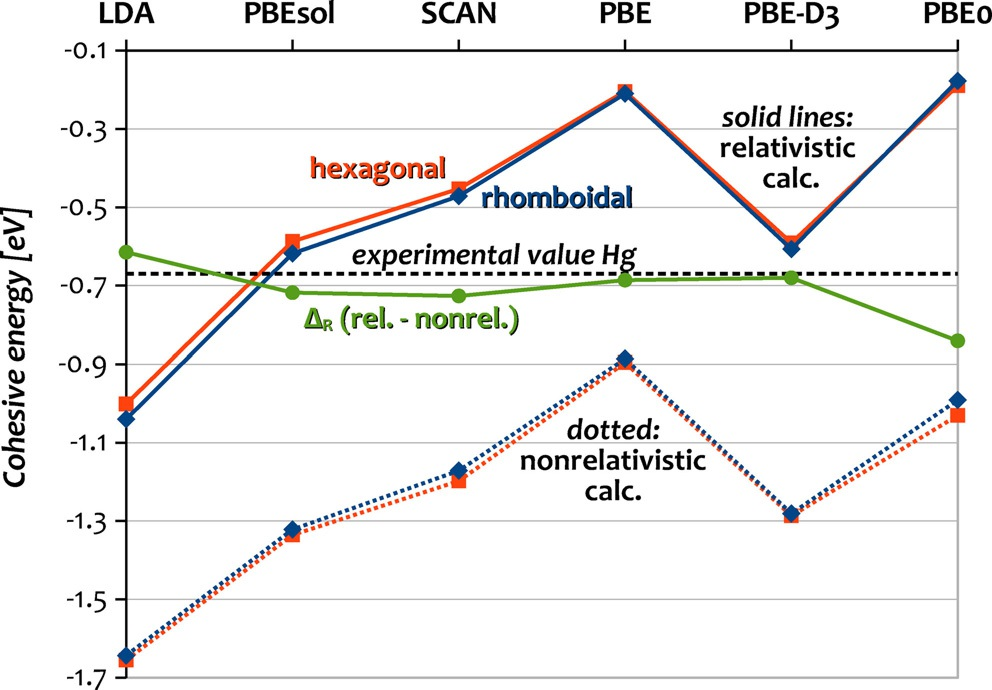
Calculated cohesive energies of hexagonal close‐packed and rhomboidal Hg with various density functionals in the relativistic and non‐relativistic picture as well as their difference Δ_R_ *E*_coh_.

A central quantity in this respect is the cohesive energy *E*_coh_ of the solids (also referred to as atomization energy), which we calculated at the relativistic and non‐relativistic level for Hg with various functionals and compare it to the experimental value in Figure 1. Inspection shows a drastic variation in the cohesive energies with the employed density functional, whereas the (absolute) relativistic change Δ_R_ shown in green remains very similar. This has already been pointed out by Steenbergen and co‐workers.^[8]^ Since PBEsol provides the best agreement for structural parameters and cohesive energy of Hg as well as consistent results for the other Group 12 elements (Table 1), we have selected it for this study. Also, from a theoretical point of view, PBEsol is well suited for the description of metallic systems. However, for the dispersion‐bound noble liquid Cn,^[31]^ the suitability of PBEsol is questionable. Despite its better agreement for the cohesive energy compared to a high‐level coupled‐cluster reference (−8 % or *λ*=1.08 for PBEsol compared to +20 % or *λ*=0.80 for dispersion‐corrected PBE‐D3),^[15]^ only dispersion‐corrected DFT can provide the correct asymptotic run of the inter‐atomic potential‐energy curve. Recently, it has been demonstrated for the related case of Og that the asymptotic run exerts a considerable influence on the calculation of phase transitions, in particular, the MP (see Figure 2 in ref. [38]). Thus, we employ the PBE‐D3 functional for Cn and compare the results to previous results obtained with PBEsol.^[31]^ Note that the parameters for DFT‐D3 for elements 112–118 have been published only recently.^[30]^

**Table 1:** Cohesive energies (*E*_coh_, in eV) of all Group 12 elements with spin–orbit (SOR), scalar (SR) and non‐relativistic (NR) DFT/PBEsol, and for Cn also PBE‐D3.^[a]^

Element	Erefcoh	ESORcoh	*λ*	ESRcoh (ΔSORSR )	ENRcoh (Δ_R_)	ENRcoh (*λ*Δ_R_)
Zn, *hcp*	−1.350	−1.572	0.859	−1.570 (0.002)	−1.661 (−0.089)	−1.426 (−0.076)
Cd, *hcp*	−1.169	−1.178	0.985	−1.169 (0.009)	−1.445 (−0.267)	−1.423 (−0.263)
Hg, *rho/hcp*	−0.670	−0.618	1.084	−0.546 (0.072)	−1.336 (−0.718)	−1.448 (−0.778)
Cn, *hcp*	−0.376	−0.349	1.078	−0.298 (0.078)	−1.333 (−1.043)	−1.436 (−1.124)
Cn, *hcp*, PBE‐D3	−0.376	−0.472	0.796	–^[b]^	–^[b]^	–^[b]^

[a] All structures are hexagonal close‐packed (*hcp*) except for relativistic Hg, which is rhomboidal (*rho*). *λ* refers to the ratio Erefcoh /ESORcoh where Erefcoh are experimental cohesive energies (ref. [6]) for Zn‐Hg and a coupled‐cluster reference for Cn.^[45]^ Δ_R_ provides the difference between the SOR and NR results. The last column provides *λ*‐scaled NR results (this corrects for the deviation of the relativistic result from the reference, see discussion). [b] PBE‐D3 parameters at the SR or NR level of theory for the dispersion correction are not available at this point.

**Figure 2:**
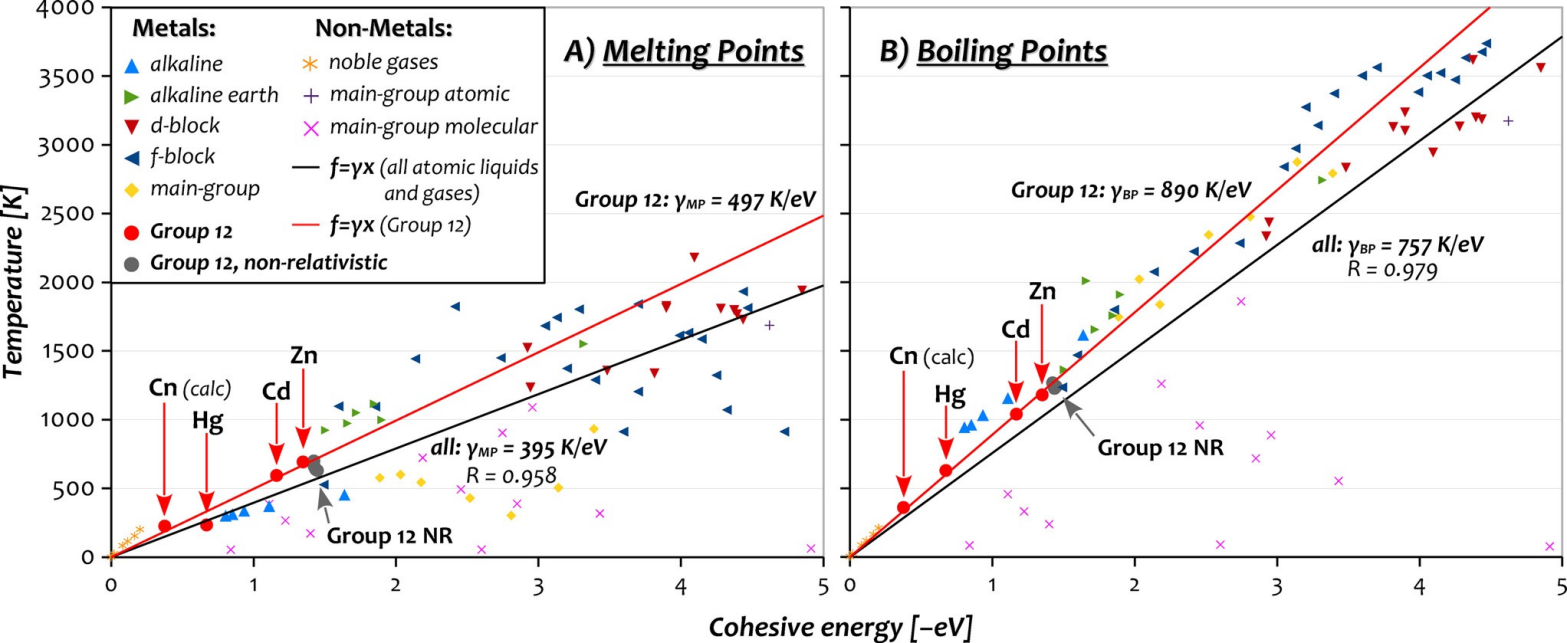
Melting and boiling points for the elements of the periodic table (data taken from refs. [6, 37]) showing a linear regression with forced ordinate intersect. Although we show only data up to 4000 K and −5 eV to better display the regime relevant for Group 12, all elements (not shown are the most strongly bound d‐block metals, C and B) are included in the fit. A spreadsheet with all data is provided in the SI.

An important observation for the cohesive energies of the Group 12 elements provided in Table 1 is that the relativistic values decrease fast with increasing nuclear charge. In contrast, the corresponding non‐relativistic values are more similar at −1.45±0.21 eV, and become virtually indistinguishable if we correct for the deviation of the calculated relativistic values from the reference value (λENRcoh =−1.43±0.02 eV). It is well known that phase‐transition temperatures (*T*_pt_) and, in particular, the BP strongly correlates with *E*_coh_ (excluding molecular gases, where the lattice energy is the relevant quantity).^[39]^ To illustrate this, we have plotted the MPs and BPs across the periodic table against the respective cohesive energies in Figure 2. Linear regression for all elements with non‐molecular gases, that is, MP/BP=*γE*_coh_, provides characteristic slopes *γ*_MP_=395 K eV^−1^ and *γ*_BP_=757 K eV^−1^ with residuals close to unity (MP: 0.96, BP: 0.98), confirming a strong correlation.

Exploiting this empirical relation, we can obtain a first estimate for the impact of relativistic effects on the transition temperatures from the (*λ*‐scaled) relativistic change of the cohesive energy, i.e., Δ_R_ *T*_pt_=*γ*_pt_ *λ*Δ_R_ *E*_coh_. Note that we use here the *λ*‐scaled difference to be consistent with our *λ*‐scaled free‐energy approach. For the example of Hg, based on the PBEsol cohesive energies in Table 1 (*λ*Δ_R_ *E*_coh_=0.78 eV) and the global slope (black line in Figure 2 A), this provides a relativistic change of Δ_R_MP=−310 K, and −390 K when using the higher slope of the fit for Group 12 (red line). For the BP, we find Δ_R_BP=−590 K or −695 K, respectively. We note that the shift of the MP is large compared to previously reported values based on a direct first‐principles simulation of the phase transition with PBEsol. In contrast it nicely agrees with the shift calculated in the same study using the local density approximation (LDA).^[8]^ In general, it is surprising that the shift reported for LDA is larger than the one reported for PBEsol since the Δ*E*_coh_ reported here and in ref. [8] are very similar or even slightly smaller with LDA (cf. Figure 1).

**Figure 3:**
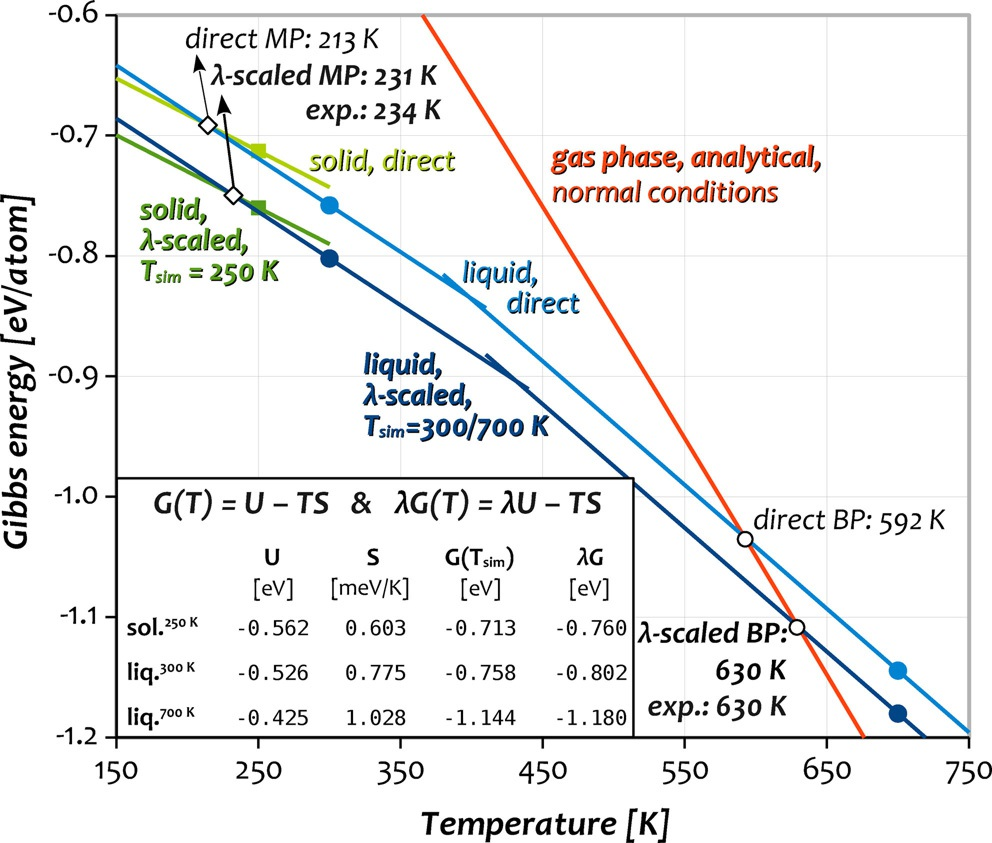
Plot of the linearly extrapolated Gibbs energy of Hg as a function of temperature with and without *λ*‐scaling applied. The kink in the free‐energy curve of the liquid shows the limitations of the linear extrapolation over several hundred K, and why it was necessary to conduct two separate calculations for Hg.

To resolve this inconsistency and test the empirical relationship—which has its limitations but can be justified for a Lennard‐Jones‐type of interaction[^40^, ^41^, ^42^]—we conducted a comprehensive study of the phase transitions of all Group 12 elements with a recently developed incremental free‐energy‐based approach. To pin down the MPs and BPs, we calculate Gibbs energies for the liquid and solid phases through thermodynamic integration (TDI) and incrementally refine them to a precision of 1 meV/atom using thermodynamic perturbation theory (TPT). For the solids, we begin from the ideal crystal at the respective equilibrium volume, add vibrational (phonon) contributions in the harmonic approximation, followed by anharmonic effects via thermodynamic integration (TDI). Eventually, we include spin–orbit coupling and converge the numerical accuracy via TPT. This is a modified variant of the UP‐TILD approach, which has been pioneered by Neugebauer and co‐workers[^43^, ^44^] and further developed by us to include relativistic effects.[^31^, ^45^] For the liquids, we start from a non‐interacting reference at the liquid equilibrium volume. As suggested by Kresse, we integrate along the interacting strength *λ* to the scalar‐relativistic DFT liquid,^[46]^ and subsequently include SO coupling and converge the numerical parameters via TPT.[^31^, ^47^] Having obtained the Gibbs energies, we locate their intersection by linear extrapolation as illustrated in Figure 3. A detailed description of the approach is provided in the Supporting Information (SI).

An important step in the approach is the so‐called *λ*‐scaling, which allows for correcting the Gibbs energies calculated at the DFT level for systematic deviations of the density functional (see Table 1 and Figure 3), thereby enabling a consistent comparison between elements. For this, the internal energy *U* contained in *G* and calculated at the DFT level is scaled with *λ*, as illustrated in Figure 3 (bright vs. dark lines). To provide an illustrative explanation in the context of Figure 2, *λ*‐scaling exploits the linear relationship between MP/BP and *E*_coh_ by shifting the predicted transition temperature along the line intersecting the calculated position of the element and the origin (i.e., the calculated slope) to match a reference cohesive energy on the *y*‐axis. In other words, the scaling allows to combine the slope (reflecting the nature of the element) from a DFT calculation with the interaction strength (reflected in *E*_coh_) from a high‐level calculation or an experiment. A detailed explanation including a formal proof in the classical Born–Oppenheimer picture can be found in refs. [31, 47]. Note that the same scaling is applied to the non‐relativistic results (see Table 1, last column). This can be motivated considering the small differences between relativistic and non‐relativistic results for Zn, which exhibits the largest systematic over‐binding and thus the largest effects of the scaling.

Eventually, linear extrapolation of the scaled Gibbs energies of the solid and liquid to their intersection as shown in Figure 3 provides the MPs. For this, it is important that both Gibbs‐energy calculations are conducted reasonably close to the intersection point, since the linear extrapolation, or in other words, the neglect of the temperature dependence of S, leads to significant errors over large temperature ranges. To obtain the BPs, we locate the intersection between the *λ*‐scaled and linearly extrapolated Gibbs energy of the liquid and an analytically modeled ideal gas.^[48]^ This recently presented approach for the calculation of BPs has been shown to provide accurate normal BPs for a representative set of elements with a mean deviation of<2 %.^[47]^

**Figure 4:**
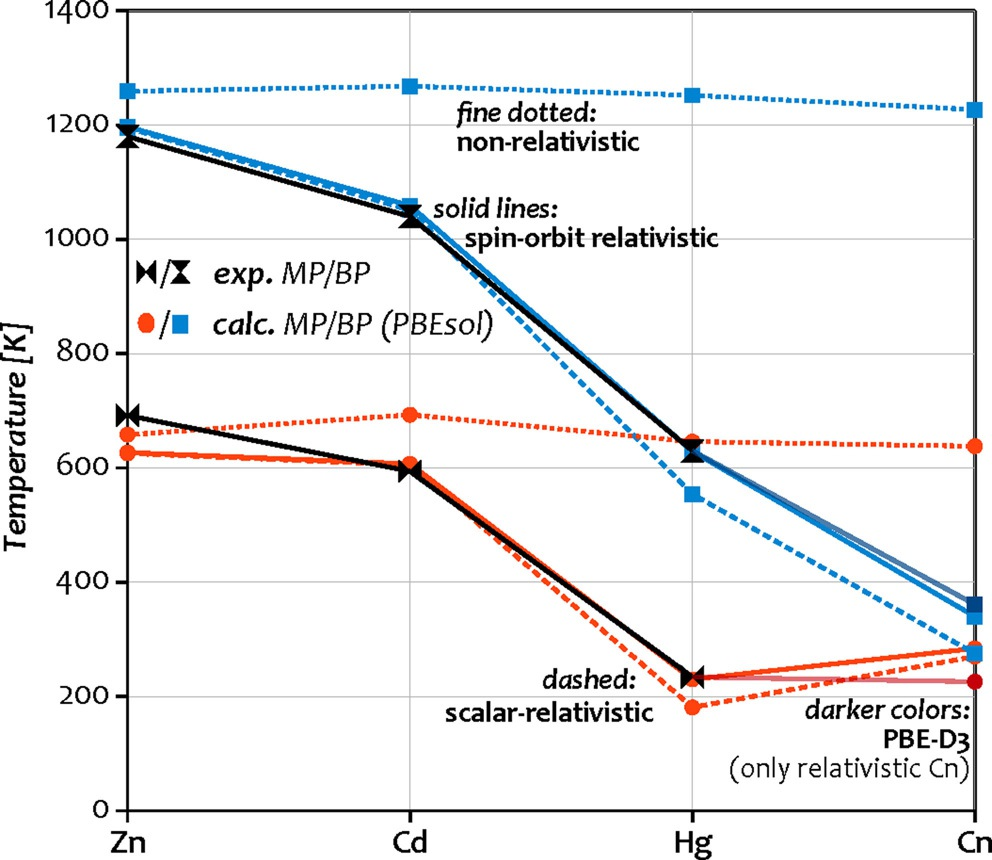
Experimental (black) and calculated MPs (orange) and BPs (blue) of the Group 12 elements.^[6]^ Spin–orbit relativistic calculations shown as solid lines, scalar‐relativistic results dashed, and non‐relativsitic results as dotted lines. All shown results are *λ*‐scaled and obtained with the PBEsol functional. For Cn, PBE‐D3 results are shown in darker colors. Lines serve only to guide the eye.

The MPs and BPs produced by this approach for Group 12 show good agreement with experimental values, as evident from Figure 4. Numbers are given in Table 2. A statistical evaluation shows the MPs to exhibit a slightly larger error than the BP, in particular for Zn (−8.4 % compared to 3.7 % and −3.0 % for Cd and Hg, respectively). This is perhaps not too surprising since the Gibbs‐energy curves of the solid and liquid run almost parallel near the MP (see Figure 3), such that the crossing point is about ten times more sensitive to errors (approx. 10 K meV^−1^) than the BP (approx. 1 K meV^−1^). Accordingly, calculated BPs are within 2 % of the experimental values, that is, 1.4 % deviation for Zn, 1.8 % for Cd, and no significant deviation for Hg. Comparing the direct results to *λ*‐scaled ones reveals a distinctly larger mean absolute deviation (MAD) for the unscaled MP (7.5 % vs. 5.0 %) and even more so for the BP (7.9 % vs. 1.1 %). Altogether, this convincingly demonstrates that the free‐energy‐based approach provides accurate absolute phase‐transition temperatures for the experimentally known Group 12 elements, which gives us confidence that our predicted values for Cn as well as relativistic shifts are also reliable.

**Table 2:** Experimental and calculated MPs and BPs of all Group 12 elements.^[a]^

Element	Exp.	SOR	SR	NR	Δ_R_
**melting points**					
Zn	694	635	635	658	−23
Cd	594	616	614	678	−62
Hg	234	231	181	648	−414
Cn (PBEsol)^[b]^	–	284	270	643	−359
Cn (PBE‐D3)	–	226	–^[c]^	–^[c]^	−417^[d]^
					
**boiling points**					
Zn	1180	1197	1195	1259	−79
Cd	1038	1060	1052	1268	−208
Hg	630	630	557	1236	−634
Cn (PBEsol)^[b]^	–	340	275	1227	−887
Cn (PBE‐D3)	–	361	–^[c]^	–^[c]^	−866^[d]^

[a] Calculated values are *λ*‐scaled and reported for spin–orbit (SOR), scalar (SR) and non‐relativistic (NR) DFT/PBEsol, and for Cn also SOR‐DFT/PBE‐D3(BJ). SR calculations employ the volume calculated at the SOR level. Errors are omitted for the sake of brevity. They amount to ±15 K in the MP, and ±5 K in the BP. [b] PBEsol data for the MP taken from ref. [45]. Values for the BP are recalculated from the provided free energies with the corrected *λ*‐scaling approach presented in ref. [47] and *λ*=1.078. [c] PBE‐D3 uses SOR parameters for D3 and thus cannot provide consistent SR or NR results. [d] With respect to the NR PBEsol result.

For Cn, the calculation with PBE‐D3 provides an MP of 226±10 K—distinctly lower than our previous PBEsol‐based estimate of 284±10 K. While this further confirms Cn's liquid aggregate state, it does not alter any of the previous conclusions about its nature.^[31]^ Moreover, this result shows that a physically correct description of dispersion is important for the phase transitions, particularly for the MP of non‐insulators and semiconductors. As such, it is consistent with a previous study for Og, for which the difference between PBEsol and PBE‐D3 is even more pronounced. A detailed analysis including a comparison of the inter‐atomic potential shapes is presented in ref. [38]. In further analogy to Og, the influence on the BP of Cn is much smaller, as evident from the BPs of 361 K (PBE‐D3) and 340 K (PBEsol), see also discussion in ref. [47].

Turning to the impact of relativistic effects, Figure 4 shows a significant influence of spin–orbit effects that is largest for Hg and a game‐changing impact of scalar‐relativistic effects. The total relativistic shift of the MP/BP is notable already for Zn with −23/−79 K, increases substantially for Cd to −62/−208 K, and once more for Hg to −414/−608 K. For Cn, the shift in the MP is comparable to Hg with −359 K (PBEsol) or −417 K (PBE‐D3 vs. NR PBEsol), while that of the BP increases to almost −900 K. The reason for the larger impact of spin–orbit effects on the MP of Hg compared to Cn lies in the size of the respective contributions: While for Cn, both phases are stabilized about equally (−58 meV/atom and −56 meV/atom for solid and liquid), the stabilization is distinctly larger for the solid (−74 meV/atom vs. −66 meV/atom) in Hg. In general, we note that the observed downward trends in the MPs and BPs within Group 12 are entirely absent in the non‐relativistic picture, consistent with the evolution of the cohesive energies. Our free‐energy calculations generally agree with large empirical estimates for the relativistic change in the MP of Hg (as well as the other Group 12 elements) of −(310–390) K, and calculated shifts are actually slightly larger. The reason for this can be understood by inspection of Figure 2: While relativistic Hg falls out of line with the other Group 12 elements and exhibits a distinctly smaller slope akin to the main‐group metals (yellow diamonds, *γ*_MP_=212 K eV^−1^), non‐relativistic Hg resembles the lighter Group 12 elements, which attain a much larger slope (*γ*_MP_=497 K eV^−1^). Hence, although Hg does in contrast to Cn remain a metal, its nature reflected in the slope is altered by relativistic effects, such that a hypothetical line connecting Hg and NR Hg in Figure 2 A has an even larger slope (*γ*_MP_=528 K eV^−1^) and does not intersect the origin.

Although the large predicted change in the MP of Hg is in good agreement with the LDA‐based estimate of Steenbergen and co‐workers, it is more than twice the value of −160 K calculated for PBEsol.[^8^, ^49^] Detailed inspection of this issue revealed that this is most likely due to methodological issues, specifically a lacking convergence of the *k*‐point grid. A detailed analysis of this aspect is provided in the SI.

Having settled the issues concerning the absolute size of relativistic effects in Group 12, we move on to ask a more general question: What is the reason for such large relativistic effects in the MPs and BPs of the Group 12 metals? As pointed out by Desclaux and Pyykkö, relativistic effects are enhanced in Group 11,^[50]^ and this is also the case in Group 12.^[21]^ The reason lies in the subtle interplay of shell‐structure effects, which includes the direct relativistic contraction of the ns orbital and the successive filling of the underlying soft (n−1)d shell undergoing relativistic expansion (indirect relativistic effects).[^11^, ^51^] Concerning phase transitions specifically, the MP/BP=*γE*_coh_ relation shows that the large changes we see are mostly due to the scale of units we choose for the temperature (the inverse Boltzmann constant is k-1B =11604 K eV^−1^) and the large relativistic change of the cohesive energy. For example, a change of 1 eV in the cohesive energy results in a change of about 400 K in the MP and 760 K in the BP, cf. Figure 2.

Taking a look at the neighboring Group 11, we note that relativistic effects *stabilize* Au as evident from Δ_R_=1.08 eV (calculated with PBEsol, in good agreement with previous studies),^[52]^ which translates into an even larger relativistic change of opposite sign (compared to Group 12), that is, an *increase* of the MP (Δ_R_MP=+425 K) and BP (Δ_R_BP=+820 K), and distinctly smaller changes for Ag (Δ_R_=0.28 eV, Δ_R_MP=+110 K, Δ_R_BP=+210 K). In relative terms, however, the effects on the MPs and BPs of the Group 11 elements are distinctly smaller than those for Group 12 and less prominent since they do not affect the aggregate state at ambient conditions.

As a second example, we discuss the low melting point of lead (Pb) of 600.6 K, which was convenient for many applications since ancient times. In analogy to the Group 12 elements, relativistic effects destabilize solid Pb by as much as Δ_R_ *E*_coh_=1.25 eV, according to Hermann and co‐workers (SOR‐DFT/PW91).^[53]^ This translates into a relativistic change of the MP of Δ_R_MP=−495 K, and of the BP of Δ_R_BP=−950 K for Pb, and is thus substantially larger than for Hg. The perhaps even more interesting case in Group 14 is flerovium (Fl). Here, the large relativistic spin–orbit splitting of the partially occupied 7p shell leads to a pseudo‐closed‐shell configuration, resulting in a huge Δ_R_ *E*_coh_ of 3.4 eV.^[53]^ This presumably leads to the perhaps largest relativistic shifts for any element of Δ_R_MP=−1330 K and Δ_R_BP=−2540 K. Based on the calculated absolute value for the relativistic cohesive energy of 0.5 eV,^[53]^ we can estimate a MP of 200 K and a BP of 380 K, indicating that Fl is a liquid similar to Cn at ambient conditions, as originally proposed by Pitzer.^[29]^ However, these considerations should be taken with a grain of salt since they are all based on a DFT calculation of the cohesive energy of Fl that is—in contrast to Cn—not backed by any high‐level calculation or experiment, and because they use fixed global slopes (black lines in Figure 2, a fit for Group 14 yields similar values of 416 K eV^−1^ and 684 K eV^−1^). However, as observed for Hg and Cn, it can be expected that the characteristic slope may vary strongly between relativistic and non‐relativistic Fl since the latter changes from a metal to an insulator.

## Summary

We have reported first‐principles free‐energy calculations for the solid and liquid phases of all Group 12 elements at relativistic and non‐relativistic levels. This allowed us to pin down their melting and boiling points (MPs and BPs) in good agreement with experimental values for Zn, Cd, and Hg (MAD= 5 % for MPs and 1 % for BPs), and to confirm the previously predicted liquid nature of Cn with dispersion‐corrected DFT (MP: 226 K, BP: 360 K with PBE‐D3). Calculations in the non‐relativistic limit were found to provide surprisingly similar MPs (650±30 K) and BPs (1250±20 K) for all Group 12 elements, suggesting that periodic trends in their phase transitions are exclusively due to relativistic effects. We discussed these results as well as deviating earlier predictions for Hg^[8]^ in the context of an empirical near‐linear relation between phase‐transition temperatures and cohesive energies. Since the latter are very similar for the Group 12 elements in the non‐relativistic limit with −1.43±0.02 eV, the empirical relation was found to nicely agree with the phase‐transition temperatures predicted from the free‐energy calculations.

Having confirmed the general validity of the empirical relation for the heavy elements, we used it to rationalize and predict relativistic effects for the related Groups 11 and 14. In general, such approximate linear relationships are very convenient for the future predictions of relativistic effects in phase transitions of many other heavy elements within the periodic table.

## Conflict of interest

The authors declare no conflict of interest.

## Supporting information

As a service to our authors and readers, this journal provides supporting information supplied by the authors. Such materials are peer reviewed and may be re‐organized for online delivery, but are not copy‐edited or typeset. Technical support issues arising from supporting information (other than missing files) should be addressed to the authors.

Supplementary

Supplementary

Supplementary
