# Supplementary material for: Exclusively Relativistic: Periodic Trends in the Melting and Boiling Points of Group 12
Source: Angew Chem Int Ed Engl. 2021 Feb 26;60(14):7703–9. doi: 10.1002/anie.202100486 (PMC8048430; doi:10.1002/anie.202100486)
Supplement: Supplementary file 1 — Supplementary [file ANIE-60-7703-s001.pdf]

Angewandte Chemie
Eine Zeitschrift der Gesellschaft Deutscher Chemiker

Supporting Information

Exclusively Relativistic: Periodic Trends in the Melting and Boiling Points of Group 12

Jan-Michael Mewes and Peter Schwerdtfeger*

anie_202100486_sm_miscellaneous_information.pdf
anie_202100486_sm_miscellaneous_information.xlsx
anie_202100486_sm_miscellaneous_information.zip

Author Contributions

J.M. Data curation: Lead; Investigation: Lead; Methodology: Lead; Visualization: Lead; Writing – original draft: Lead; Writing – review & editing: Lead
P.S. Conceptualization: Lead; Writing – review & editing: Supporting.

Supplementary Information

[1]

[2]

CONTENTS

Here we provide additional technical details for the calculations, as well as a detailed description and breakdown of the free-energy calculations.

TECHNICAL DETAILS

All calculations are carried out with the VASP 5.4.4 program package.[1–3] Core-electrons are modeled using the projector-augmented wave (PAW) approach of Joubert and Kresse with the default PBE-based PAWs from the VASP library for Zn, Cd and Hg.[4, 5] Non-relativistic PAWs were obtained for Zn, Cd, and Hg by repeating the fitting procedure used for the default scalar-relativistic PBE-PAWs (12 valence electrons, 2 projectors per shell) based on a non-relativistic all-electron calculation but with otherwise identical parameters. The generated NR PAWs are available upon request. For Cn, small-core PAWs (20 valence electrons) from a previous work were used.[6, 7]. Some important parameters of the used PAWs are collected in Table I.

TABLE I. Valence-projector energies and `ENMAX` value (in eV) for the default relativistic and non-relativistic PBE-PAW potentials used in and made for this study. NR PAWs for Zn, Cd, and Hg use the exact same parameters as the default ones (12 valence electrons). The PAWs for Cn are taken from a previous study and include the s and p semi-core levels to a total of 20 valence electrons.

element	s projectors		p projectors		d projectors		`ENMAX`
Zn	−6.0	1.2	−2.7	13.2	−10.1	−11.5	277
Zn NR	−5.8	1.8	−2.7	13.4	−10.6	−11.9	277
Cd	−5.3	−0.5	−2.7	8.6	−12.6	−14.0	275
Cd NR	−5.7	−1.6	−2.7	8.4	−11.7	−13.1	274
Hg	−6.8	−4.0	−2.7	8.1	−9.9	−11.2	233
Hg NR	−5.3	−0.4	−2.7	8.7	−12.1	−13.5	234
Cn	−136.9	−9.4	−60.7	−1.4	−8.0	−9.4	292
Cn NR	−78.6	−4.9	−52.6	−1.4	−12.3	−13.7	244

Liquid and solid phases are represented by 64-atom configurations, except for liquid Hg, where we additionally consider a 121-atom configuration with very similar results. Previous studies have confirmed that these atom numbers provide numerically converged results.[6, 8] The influence of electronic entropy is accounted for through Fermi-smearing of the populations.[9]

Two reference levels of theory are used for each element. One with reduced accuracy that allows for efficient sampling of the configuration space, and another one to obtain the final numerically converged energies. The lower reference level of theory exploits reduced global precision (`PREC = NORMAL`) and an energy-convergence of 10^{-4} eV (`EDIFF = 1E-04`) to accelerate the simulations and in in general scalar/non-relativistic (`LSORBIT = FALSE`). Liquid simulations conducted at this level use the Nose-Hover thermostat with an `SMASS` parameter of 2-4, solid simulations employ a Langevin thermostat with a `LANGEVIN_GAMMA` value of 3. The higher reference level uses (`PREC = ACCURATE`), an energy-convergence of 10^{-6} eV (`EDIFF = 1E-06`), and explicitly includes spin-orbit coupling in the valence space (`LSORBIT = TRUE`, if applicable). The element specific parameters like kinetic-energy cut-offs, k-point grids, and timesteps for the simulations are collected in Tab. II.

TABLE II. Element-specific parameters for the calculations (identical for NR, SR and SOR levels). The lower reference level used in the simulations (TDI) and the higher reference level for single-point energies (TPT) are given in the form (TDI→TPT). The timestep used in the normal simulations is given last, followed by the shorter timestep used in the simulations near the non-interacting limit (liquid only).

element (phase T_{sim})	cut-off [eV]	k-grid	timestep [fs]
Zn (solid 700 K)	$300 \rightarrow 600$	$2^3 \rightarrow 4^3$	3
Zn (liquid 1000 K)	$300 \rightarrow 600$	$2^3 \rightarrow 4^3$	3 (1)
Cd (solid 500 K)	$300 \rightarrow 600$	$2^3 \rightarrow 4^3$	4
Cd (liquid 1000 K)	$300 \rightarrow 600$	$2^3 \rightarrow 4^3$	4 (2)
Hg (solid 250/500 K)	$250 \rightarrow 500$	$2^3 \rightarrow 5^3$	6
Hg (liquid 300/700 K)	$250 \rightarrow 500$	$2^3 \rightarrow 5^3$	6 (2)
Cn (solid 200 K)	$400 \rightarrow 600$	$2^3 \rightarrow 5^3$	8
Cn (liquid 250/300 K)	$400 \rightarrow 600$	$2^3 \rightarrow 5^3$	8 (2)

FREE-ENERGY CALCULATIONS

Solid − The free energy of the solids is calculated in four main steps, in the first of which the electronic energy

TABLE III. Incremental contributions to the Gibbs energy of Zn using the PBEsol functional in eV/atom. The internal energy U is the average of a canonical MD simulation and includes the TPT corrections.

step (level)	contrib.	total G_s	error
**Zn/solid/700 K/relativistic**			
electronic energy	−1.5754		
harmonic crystal	−0.2808	−1.8562	
anharm. via TDI	0.0020	−1.8542	
TPT cut-off & prec.	0.0019	−1.8523	
TPT k-points	0.0184	−1.8339	
TPT spin-orbit	−0.0017	−1.8356	±0.0005
internal energy U		−1.3783	±0.0005
entropy S [meV/K]		0.6533	
**Zn/solid/700 K/non-relativistic**			
electronic energy	−1.6700		
harmonic crystal	−0.2726	−1.9427	
anharm. via TDI	0.0002	−1.9424	
TPT cut-off & prec.	0.0019	−1.9405	
TPT k-points	0.0191	−1.9214	±0.0005
internal energy U		−1.4704	±0.0005
entropy S [meV/K]		0.6443	
**Zn/liquid/1000 K/relativistic**			
ideal gas @ liq. volume	−1.0185		
TDI to DFT liq.	−1.0695	−2.0880	
TPT cut-off & prec.	0.0022	−2.0859	
TPT k-points	−0.0047	−2.0905	
TPT spin-orbit	−0.0016	−2.0921	±0.001
internal energy U		−1.2067	±0.001
entropy S [meV/K]		0.8854	
**Zn/liquid/1000 K/non-relativistic**			
ideal gas @ liq. volume	−1.0202		
TDI to DFT liq.	−1.1472	−2.1674	
TPT cut-off & prec.	0.0019	−2.1655	
TPT k-points	−0.0049	−2.1704	±0.001
internal energy U		−1.2879	±0.001
entropy S [meV/K]		0.8825	

TABLE IV. Incremental contributions to the Gibbs energy of Cd using the PBEsol functional in eV/atom. The internal energy U is the average of a canonical MD simulation and includes the TPT corrections.

step (level)	contrib.	total G_s	error
**Cd/solid/500 K/relativistic**			
electronic energy	−1.1707		
harmonic crystal	−0.2239	−1.3946	
anharm. via TDI	0.0025	−1.3921	
TPT cut-off & prec.	−0.0006	−1.3927	
TPT k-points	0.0095	−1.3832	
TPT spin-orbit	−0.0089	−1.3921	±0.0005
internal energy U		−1.0460	±0.0005
entropy S [meV/K]		0.6922	
**Cd/solid/500 K/non-relativistic**			
electronic energy	−1.4562		
harmonic crystal	−0.2045	−1.6607	
anharm. via TDI	0.0004	−1.6603	
TPT cut-off & prec.	−0.0007	−1.6610	
TPT k-points	0.0170	−1.6440	±0.0005
internal energy U		−1.3136	±0.0005
entropy S [meV/K]		0.6609	
**Cd/liquid/1000 K/relativistic**			
ideal gas @ liq. volume	−1.1240		
TDI to DFT liq.	−0.7179	−1.8419	
TPT cut-off & prec.	−0.0004	−1.8423	
TPT k-points	−0.0051	−1.8474	
TPT spin-orbit	−0.0083	−1.8557	±0.001
internal energy U		−0.8498	±0.001
entropy S [meV/K]		1.0059	
**Cd/liquid/1000 K/non-relativistic**			
ideal gas @ liq. volume	−1.1267		
TDI to DFT liq.	−0.9456	−2.0723	
TPT cut-off & prec.	−0.0006	−2.0729	
TPT k-points	−0.0039	−2.0768	±0.001
internal energy U		−1.0869	±0.001
entropy S [meV/K]		0.9898	

and ionic frequencies of the super-cells are calculated at their respective equilibrium volumes either non- or scalar-relativistically. For the ionic frequencies, the numerical precision is increased by setting `Precision=Accurate` and `LREAL=False` in the `INCAR` file to avoid aliasing errors with the finite-differences method. To make these calculations more accurate, four displacements are considered for each atom in each direction (`NFREE=4`).

Secondly, using the previously determined force-constants, the vibrational contributions to the free energy are calculated in the harmonic approximation. These calculations are carried out with the Phonopy program using a very fine k-point mesh (16^3).[11]

Thirdly, thermodynamic integration from the harmonic to the (non- or scalar-relativistic) DFT solid is carried out by interpolating between harmonic and DFT forces. The integral is evaluated using a Gauss-Legendre three-point rule with at least 5000 steps time at each point (1000 steps equilibration, 4000+ steps productive). This provides the anharmonic correction to the free energy of the harmonic crystal, which is the main source of errors for the free energy of the solid with a statistical uncertainty of $0.1 - 0.4$ meV.

Finally, the difference between the low and high reference levels (spin-orbit coupling, k-point mesh, kinetic-energy cut-off, and increased numerical precision, see Tab. II) is accounted for using TPT, *i.e.*, by recalculating the electronic energy for at least 10 frames at both

TABLE V. Incremental contributions to the Gibbs energy of Hg using the PBEsol functional in eV/atom. The internal energy U is the average of a canonical MD simulation and includes the TPT corrections.

step (level)	contrib.	total G_s	error
**Hg/solid/250 K/relativistic**			
electronic energy		−0.5612	
harmonic crystal	−0.0859	−0.6471	
anharm. via TDI	−0.0003	−0.6474	
TPT cut-off & prec.	0.0001	−0.6473	
TPT k-points	0.0080	−0.6393	
TPT spin-orbit	−0.0736	−0.7128	±0.0005
internal energy U		−0.5622	±0.0005
entropy S [meV/K]		0.6027	
**Hg/solid/500 K/non-relativistic**			
electronic energy		−1.3395	
harmonic crystal	−0.2510	−1.5904	
anharm. via TDI	0.0006	−1.5898	
TPT cut-off & prec.	−0.0005	−1.5902	
TPT k-points	0.0130	−1.5974	±0.0005
internal energy U		−1.2015	±0.0005
entropy S [meV/K]		0.7521	
**Hg/liquid/300 K/relativistic**			
ideal gas @ liq. volume		−0.3134	
TDI to DFT liq.	−0.3798	−0.6932	
TPT cut-off & prec.	0.0005	−0.6927	
TPT k-points	0.0005	−0.6923	
TPT spin-orbit	−0.0658	−0.7580	±0.001
internal energy U		−0.5256	±0.001
entropy S [meV/K]		0.7694	
**Hg/liquid/700 K/relativistic**			
ideal gas @ liq. volume		−0.8114	
TDI to DFT liq.	−0.2684	−1.0799	
TPT cut-off & prec.	−0.0010	−1.0809	
TPT k-points	0.0004	−1.0805	
TPT spin-orbit	−0.0639	−1.1444	±0.001
internal energy U		−0.4248	±0.001
entropy S [meV/K]		1.0028	
**Hg/liquid/700 K/non-relativistic**			
ideal gas @ liq. volume		−0.8142	
TDI to DFT liq.	−0.9322	−1.7464	
TPT cut-off & prec.	0.0000	−1.7464	
TPT k-points	−0.0036	−1.7500	±0.001
internal energy U		−1.0710	±0.001
entropy S [meV/K]		0.9701	

levels, typically with several intermediate steps to check the size of each single contribution for convergence. Formally, TPT provides the free-energy difference between two Hamiltonians

$$\Delta G_{1-2} = -\frac{1}{\beta} \ln\langle e^{-\beta[U_2(\mathbf{R})-U_1(\mathbf{R})]}\rangle_1 \ , \qquad (1)$$

TABLE VI. Incremental contributions to the Gibbs energy of Cn using the PBE-D3 functional in eV/atom. The internal energy U is the average of a canonical MD simulation and includes the TPT corrections.

step (level)	contrib.	total G_s	error
**Cn/solid/200 K/relativistic**			
electronic energy		−0.4316	
harmonic crystal	−0.0914	−0.5230	
anharm. via TDI	0.0021	−0.5209	
TPT cut-off & prec.	−0.0036	−0.5245	
TPT k-points	−0.0001	−0.5246	
TPT spin-orbit	−0.0362	−0.5608	±0.0005
internal energy U		−0.4234	±0.0005
entropy S [meV/K]		0.6873	
**Cn/liquid/250 K/relativistic**			
ideal gas @ liq. volume		−0.2737	
TDI to DFT liq.	−0.2806	−0.5543	
TPT cut-off & prec.	−0.0034	−0.5577	
TPT k-points	0.0000	−0.5577	
TPT spin-orbit	−0.0355	−0.5932	±0.001
internal energy U		−0.3958	±0.001
entropy S [meV/K]		0.7898	
**Cn/liquid/300 K/relativistic**			
ideal gas @ liq. volume		−0.3362	
TDI to DFT liq.	−0.2618	−0.5979	
TPT cut-off & prec.	−0.0031	−0.6011	
TPT k-points	0.0000	−0.6011	
TPT spin-orbit	−0.0344	−0.6354	±0.001
internal energy U		−0.3802	±0.001
entropy S [meV/K]		0.8507	

where the index after the angle bracket indicates that the difference ΔU_{1-2} is evaluated for configurations generated by H_1. Instead of the exact equation, we use the second-order cumulant expansion

$$\Delta G_{1-2} \approx \langle \Delta U\rangle_1 - \frac{\beta}{2}\langle(\Delta U - \langle\Delta U\rangle)^2\rangle_1 \ , \qquad (2)$$

which is sufficiently accurate since already the second-order term is $\ll 1$ meV/atom in all cases, indicating a parallel run of the respective potential-energy surfaces.

Statistical errors are < 0.2 meV in all cases. The contributions and final Gibbs free energies of the solids are collected in Tabs. III-VI.

Liquid – The Gibbs energy of the liquid is calculated through TDI from a non-interacting reference. For this purpose the difference of the internal energies is integrated along the coupling parameter λ

$$\Delta G_{0-1} = \int_0^1 d\lambda \langle U_1(\mathbf{R}) - U_0(\mathbf{R})\rangle_\lambda \ . \qquad (3)$$

The free energy of the non-interacting reference at the

TABLE VII. Calculated atomic equilibrium volumes ($V_{\rm eq}$ in A^3/atom), corresponding densities (ρ in g/ccm) and averaged residual pressures ($\langle p \rangle$ in kBar) of all considered phases and temperatures. Calculations with spin-orbit (SOR) and non-relativistic (NR) PBEsol for Zn-Hg and PBE-D3 for Cn. Simulations are conducted at the lower reference level, while corrections for the higher reference level are calculated for 10 statistically independent snapshots from the simulation. The difference due to spin-orbit coupling $\Delta_p^{\rm SO}$ is given in the last column.

System	$T_{\rm sim}$	$V_{\rm eq}$	$\rho_{\rm calc}$	$\rho_{\rm exp}$	$\langle p \rangle$	$\Delta p^{\rm SO}$
Zn, *hcp*	700	14.83	7.32	7.1[a]	−0.04	−0.09
Zn^{nr}, *hcp*	700	15.13	7.18		0.17	–
Zn, *lqd*	1000	16.68	6.51	6.4[a]	−0.51	−0.09
Zn^{nr}, *lqd*	1000	17.00	6.39		−0.14	–
Cd, *hcp*	500	21.90	8.52	8.7[b]	−0.74	−0.97
Cd^{nr}, *hcp*	500	22.76	8.20		0.17	–
Cd, *lqd*	1000	25.15	7.42	7.5[a]	−0.83	−0.84
Cd^{nr}, *lqd*	1000	25.95	7.19		−0.07	–
Hg,[b] *rho*	250	24.33	13.7	14.2	−0.05	−10.12
Hg^{nr}, *hcp*	250	25.30	13.2		−0.56	–
Hg^{nr}, *hcp*	500	25.86	12.9		0.06	–
Hg,[b] *lqd*	300	25.11	13.3	13.5[b]	−0.65	−9.32
Hg, *lqd*	700	25.88	12.9	11.9[a]	−0.46	−8.40
Hg^{nr}, *lqd*	700	28.38	11.7		−0.18	–
Cn, *hcp*	200	34.32	13.8		−0.13	−4.51
Cn, *lqd*	250	35.59	13.3		−0.17	−4.33
Cn, *lqd*	300	35.51	13.0		−0.56	−4.23

[a] Extrapolated from the data provided in ref. [10]
[b] at ambient conditions

liquid equibrium volume is

$$G^{\rm id} = F^{\rm id} = -k_B T \ln\left(Z(\Theta, T, V, N)\right), \text{ where} \qquad (4)$$

$$Z(T,V,N) = \frac{(\Theta V)^N}{\Lambda^{3N} N!} \text{ and } \Lambda = h\sqrt{\frac{\beta}{2\pi m}} \quad . \qquad (5)$$

Here, N is the number of atoms, m their mass, V the atomic volume, and Θ the electronic degeneracy. Note that the pV term is negligible at the volume of the liquid ($\ll 1$ meV/atom) and only taken into account for the gas phase below.

Since the kinetic energy part of U_1 and U_0 is identical it cancels, and the potential part vanishes at zero interaction stength (U_0), the value of the integrand is the average internal potential energy calculated at full interaction strength $\langle U_1^{\rm pot}(\mathbf{R})\rangle$ for configurations $\mathbf{R}$ generated with reduced interaction strength (at the respective λ). This integral is evaluated using numerical quadrature in the form of a n-point Gauss-Lobatto rule, in principle requiring one NVT simulation for each value of λ. We use $n = 6$ for Zn and Cd, $n = 8$ for Hg and Cn. While most of these simulations are straightforward, the ones very close to the ideal-gas limit ($\lambda \ll 0.01$ or $< 1\%$ of the DFT forces) are tedious, whereas the simulation for the end point $\lambda = 0$ is not possible with a PAW+DFT methodology. This is because close-encounters between the (almost) non-interacting atoms lead to singularity in the energy resulting in numerical instabilities in errors in the simulations, partly resulting from overlapping core-electrons. An approach to circumvent these issues was devised and implemented by Kresse and coworkers and will be used here with slight modifications.[8]

The approach is based on substituting λ in eq. (3) with $\lambda(x) = (\frac{x+1}{2})^{1/(1-\kappa)}$, which yields

$$\Delta G_{0-1} = \frac{1}{2(1-\kappa)} \int_{-1}^{1} dx \langle U_1^{\rm pot}(\mathbf{R}) \lambda(x)^{\kappa} \rangle_x \, . \qquad (6)$$

This introduces an explicit dependency on λ in the integrand, which not only dampens the impact of the technically challenging calculations near the non-interacting limit, and eliminates the point for $\lambda = 0$. The parameter κ guides the mapping of the quadrature points between the domains. While a value close to 0 retains the original (equidistant) spacing of the Gauss-Lobatto quadrature, κ close to unity increases the density of quadrature points in the λ domain in the region close to $\lambda = 0$, where the slope of $f(\lambda)$ is the largest. Kresse and coworkers suggest $\kappa > 0.8$. In a recent work,[12] we demonstrated that smaller values can suffice and lead to much more manageable simulations. We use $\kappa = 0.7$ for all elements, having tested 0.8 for Cn (300 K) with very similar results. In analogy to the solid, TPT is used to correct and the final energies with a desired precision of ≈ 1 meV/atom, which requires about 10 snapshots from each simulation.

Gas Phase – The Gibbs energy of the gas phase is calculated for the non-interacting (ideal) gas at its equilibrium volume and ambient pressure. For a given atomic degeneracy Θ, volume $V = nkT/p$, temperature T, particle number N and mass m this is

$$G^{\rm id} = F^{\rm id} + pV = -k_B T \ln\left(Z(\Theta, T, V, N)\right) + pV \ , \qquad (7)$$

where Z is defines in eq. (5). For the gas phase, the logarithm in Z is solved using the Stirling approximation, which is sufficiently accurate since we are considering an arbitrary number of particles.

To check if the non-interacting model is sufficient, we evaluate the first virial (two-body) correction for each of the examples assuming a Lennard-Jones (12,6) potential with the parameters for the dimers provided in the introduction. This leads to the following integral

$$G^{g}_{\rm LJ} = G^{g}_{\rm id} - \frac{2\pi N^2}{V\beta} \int \left[r^2 e^{-4\epsilon\beta\left[\left(\frac{\sigma}{r}\right)^{12} - \left(\frac{\sigma}{r}\right)^{6}\right]} - 1 \right] dr \qquad (8)$$

which can be evaluated as described in ref. 13. Due to the weak interaction between isolated Zn, Cd and Hg atoms and their large R_es, the correction is repulsive and negligibly small at the respective BPs (≤ 0.1 meV/atom). At the distinctly lower BP of Cn, the virtial term is attractive and somewhat larger at -0.2 meV/atom, yet still too small to affect the calculated BP.

CALCULATIONS OF ERRORS

Statistical errors for averages taken from simulations are calculated using block-averaging. The typical block size for the solid configuration is about 50, and distinctly larger with 50-150 for the liquid depending on time-step and λ. Based on these data, we have chosen the length of the simulations to achieve a statistical error smaller than 1 meV/atom. The other significant source of errors is the linear extrapolation of $G(T)$ for the solid and liquid (cf. Fig. 3 in the main article). To mitigate these errors, we have conducted the free-energy calculations near the respective MP/BP. Altogether, this leads to an uncertainty of about 15 K in the predicted MPs and about 5 K in the BP, the latter of which is less sensitive due to the steeper intersection of the respective free-energy curves.

ORIGIN OF THE DIFFERENCE TO THE PREVIOUS ESTIMATE OF STEENBERGEN

We note that the interface-pinning method employed in ref. 14 requires much larger cells of 250 atoms (125 liquid and 125 solid atoms), which was only possible (despite using a large supercomputer) at a scalar-relativistic level with a rather coarse k-point grid ($2 \times 2 \times 1$ or effectively $2 \times 2 \times 2$ points for the solid/liquid part as these are joined third axis). In contrast, our approach allows to use smaller 64-atom solid ($4 \times 4 \times 4$ super-cell) and 64- or 121-atom liquid configurations, which in combination with the incremental corrections via TPT allows to include much finer k-point grids of up to $5 \times 5 \times 5$ as well as explicit spin-orbit coupling. Inspection of the respective TPT corrections from the $2 \times 2 \times 2$ grid employed in our simulations, which corresponds to the final level of ref. 14, to the final $5 \times 5 \times 5$ grid used here (cf. Table V) reveals a strong influence and large differences in the respective corrections for the liquid (< 1 meV/atom), relativistic solid (8 meV/atom), and non-relativistic solid phases (13 meV/atom). Although a detailed analysis of the influence this has on the predicted MP is not possible, the large differential corrections strongly suggest this as the origin of the deviation.

* janmewes@janmewes.de
† p.a.schwerdtfeger@massey.ac.nz

[1] G. Kresse and J. Hafner, Phys. Rev. B **47**, 558 (1993).
[2] G. Kresse and J. Hafner, Phys. Rev. B **49**, 14251 (1994).
[3] G. Kresse and J. Furthmüller, Phys. Rev. B **54**, 11169 (1996).
[4] P. E. Blöchl, Phys. Rev. B **50**, 17953 (1994).
[5] G. Kresse and D. Joubert, Phys. Rev. B **59**, 1758 (1999).
[6] J.-M. Mewes, O. R. Smits, G. Kresse, and P. Schwerdtfeger, Angew. Chem. Int. Ed. **58**, 17964 (2019).
[7] L. Trombach, S. Ehlert, S. Grimme, P. Schwerdtfeger, and J.-M. Mewes, Phys. Chem. Chem. Phys. **21**, 18048 (2019).
[8] F. Dorner, Z. Sukurma, C. Dellago, and G. Kresse, Phys. Rev. Lett. **121**, 195701 (2018).
[9] N. D. Mermin, Phys. Rev. **137**, A1441 (1965).
[10] M. Assael, I. J. Armyra, J. Brillo, S. V. Stankus, J. Wu, and W. A. Wakeham, J. Phys. Chem. Ref. Data **41**, 033101 (2012).
[11] A. Togo and I. Tanaka, Scr. Mater. **108**, 1 (2015).
[12] J.-M. Mewes and O. R. Smits, Phys. Chem. Chem. Phys. **22**, 24041 (2020).
[13] B. Cowan, *Topics in Statistical Mechanics*, Imperial College Press Advanced Physics Texts, Vol. 3 (Imperial College Press, 2005).
[14] K. G. Steenbergen, J.-M. Mewes, L. F. Pasteka, H. W. Gaggeler, G. Kresse, E. Pahl, and P. Schwerdtfeger, Phys. Chem. Chem. Phys. **19**, 32286 (2017).
